# Clinical Dilemma Involving Treatments for Very Low-Birth-Weight Infants and the Potential Risk of Necrotizing Enterocolitis: A Narrative Literature Review

**DOI:** 10.3390/jcm13010062

**Published:** 2023-12-22

**Authors:** Shigeo Iijima

**Affiliations:** Department of Regional Neonatal-Perinatal Medicine, Hamamatsu University School of Medicine, Hamamatsu 431-3192, Japan; siijima@hama-med.ac.jp; Tel.: +81-53-435-2312

**Keywords:** necrotizing enterocolitis, risk factor, indomethacin, corticosteroid, histamine-2 receptor blocker, doxapram, glycerin enema, antibiotics, blood transfusion, probiotics

## Abstract

Necrotizing enterocolitis (NEC) is a critical gastrointestinal emergency with substantial morbidity and mortality risks, especially for very low-birth-weight (VLBW) infants, and unclear multifactorial pathophysiology. Whether common treatments for VLBW infants increase the NEC risk remains controversial. Indomethacin (utilized for patent ductus arteriosus) offers benefits but is concerning because of its vasoconstrictive impact on NEC susceptibility. Similarly, corticosteroids used to treat bronchopulmonary dysplasia may increase vulnerability to NEC by compromising immunity and altering the mesenteric blood flow. Histamine-2 receptor blockers (used to treat gastric bleeding) may inadvertently promote NEC by affecting bacterial colonization and translocation. Doxapram (used to treat apnea) poses a risk of gastrointestinal disturbance via gastric acid hypersecretion and circulatory changes. Glycerin enemas aid meconium evacuation but disrupt microbial equilibrium and trigger stress-related effects associated with the NEC risk. Prolonged antibiotic use may unintentionally increase the NEC risk. Blood transfusions for anemia can promote NEC via interactions between the immune response and ischemia–reperfusion injury. Probiotics for NEC prevention are associated with concerns regarding sepsis and bacteremia. Amid conflicting evidence, this review unveils NEC risk factors related to treatments for VLBW infants, offers a comprehensive overview of the current research, and guides personalized management strategies, thereby elucidating this clinical dilemma.

## 1. Introduction

Necrotizing enterocolitis (NEC) profoundly affects premature infants, especially those with very low-birth-weight (VLBW), and has a prevalence of approximately 7.6% among VLBW infants and an estimated death rate of 20–30% among infants with birth weights of 501–1500 g [1]. NEC involves inflammation and necrosis of the intestinal tissue, thereby posing a significant threat. The etiology of NEC is complex, multifactorial, and not fully understood. The most typical initial signs and symptoms of NEC in a preterm infant include feeding intolerance, abdominal distention, and bloody stools after 8 to 10 days of age [2]. To assess the severity of NEC, Bell’s classification has been used. Bell’s stage I is characterized by suspected or unproven NEC, stage II comprises confirmed NEC, and stage III comprises advanced NEC. Abdominal radiography remains the standard imaging procedure for evaluating NEC; however, recently, abdominal ultrasound has been shown to be beneficial for diagnosing and managing NEC [3].

Many studies have reported the clinical and non-clinical risk factors associated with NEC, including various treatment strategies for several medical conditions of VLBW infants. Indomethacin is used for patent ductus arteriosus (PDA). Corticosteroids are used for bronchopulmonary dysplasia (BPD). Histamine-2 receptor blockers (H2 blockers) are used for gastric bleeding. Doxapram is used for apnea. Antibiotics are used for infections. Glycerin enemas are used for meconium-related conditions. Blood transfusions are used for anemia or blood loss. However, it has been suggested that these treatments are associated with an increased incidence of NEC. In contrast, probiotics have been shown to prevent NEC; however, this therapy is associated with concerns about NEC and sepsis. Therefore, the associations between these treatments and NEC are the subject of ongoing debate and research.

This review adopted a narrative approach and delved into the clinical dilemma encompassing treatments for VLBW infants, their potential to increase the NEC risk, and the challenges they pose. Furthermore, it focused on exploring alternatives and modifications to medication regimens to mitigate the risk of NEC.

## 2. Literature Research Methods

A comprehensive literature review was performed using well-established medical and scientific databases, such as PubMed, Cochrane Library, Google Scholar, ResearchGate, and Igaku Chuo Zasshi-Web for Japanese medical literature. Additionally, major internet search engines, such as Google and Yahoo!, were used. The search included animal model studies, controlled and uncontrolled studies, systematic reviews, meta-analyses, and case reports. Articles written in the English and Japanese languages were considered. No specific publication timeframes were used. Various combinations of keywords, such as “necrotizing enterocolitis”, “premature neonate”, “medication”, “adverse effect”, “indomethacin”, “corticosteroid”, “H2-blocker”, “doxapram”, “antibiotics”, “enema”, “blood transfusion”, and/or “probiotics”, were searched. The articles were screened based on their abstracts and titles, followed by a thorough examination of the full text of the selected articles. The exclusion criteria were applied to ensure relevance to NEC. Furthermore, the references of the initial articles were used to identify additional relevant literature.

## 3. Pathophysiology of NEC

**Figure 1:**
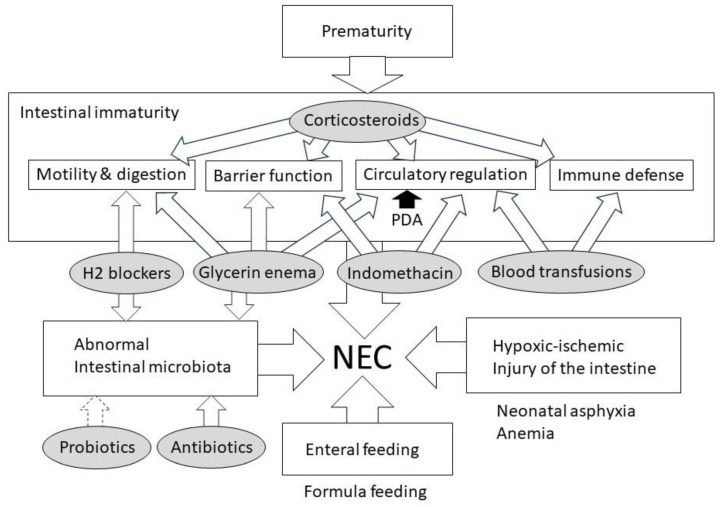
Pathogenesis of necrotizing enterocolitis and impact of treatments for very low-birth-weight infants. Abbreviations: PDA, patent ductus arteriosus; NEC, necrotizing enterocolitis; H2 blockers, histamine-2 receptor blockers.

The etiology and pathogenesis of NEC are multifactorial and have not yet been fully elucidated. Prematurity, low birth weight, formula feeding, and intestinal dysbiosis are key predisposing factors for NEC. Additionally, maternal factors such as chorioamnionitis, fetal factors such as genetic predisposition and intrauterine growth restriction, and neonatal factors such as hypoxia and PDA are known to increase the incidence of NEC (Table 1) [4,5]. Medications commonly used for VLBW infants and blood transfusions have been suggested as neonatal factors as well. The pathogenesis of NEC is mainly related to the intestinal immaturity of motility and digestion, barrier function, circulatory regulation, and immune defense, and the NEC risk is escalated by factors such as hypoxia, abnormal intestinal microbiota, enteral feeding (formula feeding), and inflammation-triggering mediators caused by ischemia–reperfusion injury to the immature gut (Figure 1) [6].

## 4. Clinical Dilemma Involving Medications and the Risk of NEC

### 4.1. Indomethacin

PDA, which is a condition that is prevalent among more than 60% of extremely premature infants [7], is significant because of its potential association with NEC, which is a condition exacerbated by reduced gastrointestinal perfusion. Non-steroidal anti-inflammatory drugs (NSAIDs), particularly indomethacin, have been used to achieve pharmacological PDA closure. However, the vasoconstrictive effect of indomethacin is associated with concerns regarding the risk of NEC (Table 2).

#### 4.1.1. Mechanisms Underlying Indomethacin and NEC Development

Indomethacin inhibits cyclooxygenase (COX) enzymes that drive prostaglandin production in the ductus arteriosus and other blood vessels [8]. By inhibiting COX enzymes, indomethacin reduces prostaglandin synthesis, constricts intestinal arteries, and compromises perfusion [8]. Furthermore, prostaglandins modulate inflammation and immune responses [9], thereby implicating that indomethacin disrupts intestinal immune homeostasis and contributes to NEC inflammation.

#### 4.1.2. Clinical Insights Regarding Indomethacin and NEC

Grosfeld et al. found a higher NEC (stage unknown) incidence (35%) among infants treated with indomethacin for PDA closure than among their untreated counterparts (13%) [10]. Fujii et al. observed intestinal perforation caused by NEC in 20% of extremely premature infants (<27 weeks of gestation) who underwent indomethacin therapy within 48 h of life [11]. In contrast, a randomized study by Schmidt et al. and a retrospective study by O’Donovan et al. showed that indomethacin therapy was not associated with the NEC (Bell’s stage ≥ II) risk [12,13]. A population study by Dollberg et al. revealed no significant effect of indomethacin on the NEC risk; however, PDA itself increased the NEC (Bell’s stage ≥ II) risk [14]. Meta-analyses collectively showed that prophylactic and therapeutic indomethacin treatments did not increase the NEC (all Bell’s stages and Bell’s stage ≥ II) risk [15,16].

#### 4.1.3. Strategies for Risk Mitigation and Exploration of Safer Alternatives

The benefits of indomethacin for PDA closure counterbalance the negative effects of perfusion. Both PDA and indomethacin decrease the intestinal blood flow. Treatment of PDA with early clinical signs results in better mitigation of the NEC risk than treatment after signs of congestive cardiac failure have been observed [17], thereby highlighting the role of PDA itself. Early indomethacin treatment reduces the NEC risk. Ibuprofen, an NSAID similar to indomethacin, effectively closes the PDA in preterm infants [18]. A Cochrane review indicated that ibuprofen is superior for reducing the risk of NEC [19]. Acetaminophen, which inhibits peroxidase rather than COX, has been proposed as an alternative to indomethacin or ibuprofen; however, its evidence is insufficient. A recent Cochrane review showed that the efficacy of paracetamol was similar to that of ibuprofen and involved a comparable risk of NEC [20].

### 4.2. Corticosteroids

Corticosteroids, such as dexamethasone and hydrocortisone, have potent anti-inflammatory properties and are used to manage various medical conditions of premature infants. Postnatal steroid therapy is primarily used to prevent or treat BPD [21]. BPD, which affects approximately 40% of extremely preterm infants, leads to significant mortality as well as respiratory and neurodevelopmental morbidity [21]. However, a crucial concern is the potential for life-threatening gastrointestinal bleeding and perforation associated with postnatal corticosteroid use [22]. Moreover, although rare, an association between postnatal steroid use and the risk of NEC has been reported (Table 2) [23]. Despite their distinct natures, NEC and intestinal perforations can share synergistic risk factors for extremely preterm infants [24]. In contrast, antenatal corticosteroid therapy for expectant preterm births is often used because of its role in fetal organ maturation and improved postnatal survival; furthermore, this therapy is correlated with a reduced incidence of NEC [25].

#### 4.2.1. Mechanisms Underlying Corticosteroids and NEC Development

Corticosteroids possess immunosuppressive attributes, mitigate inflammatory responses, and alleviate lung inflammation in premature infants with BPD. However, this immunosuppression may compromise intestinal immune defenses, rendering infants more susceptible to NEC-inducing factors. Furthermore, corticosteroids can influence intestinal motility and perfusion [26], possibly leading to a dysregulated transit time and NEC susceptibility. Additionally, an altered mesenteric blood flow caused by corticosteroid use might compromise the intestinal mucosal barrier [27], thereby elevating the bacterial translocation risk. Notably, studies have suggested that early postnatal dexamethasone use might cause an imbalance of tissue growth [28], rendering the bowel wall susceptible to other factors such as indomethacin [23].

#### 4.2.2. Clinical Insights Regarding Corticosteroids and NEC

Toll-like receptor 4 (TLR4)-mediated proinflammatory and anti-inflammatory signaling imbalances contribute to NEC development in the premature intestinal epithelium [29]. Animal studies have indicated that both indomethacin and dexamethasone increase the risk of NEC by increasing TLR4 mRNA expression in the intestinal tract [30]. A retrospective study has shown an association between glucocorticoid exposure and indomethacin exposure during the first week of life and the risk of NEC (Bell’s stage ≥ I) [23]. Cochrane reviews revealed that early (<8 days after birth) systemic corticosteroid use significantly increased the risks of gastrointestinal bleeding and perforation [31]. However, there is no conclusive evidence regarding the risk of NEC (stage unknown) with both early and late (>7 days after birth) corticosteroid use [32].

Antenatal corticosteroid therapy is associated with a decreased incidence of NEC, potentially aiding the development of an immature intestinal barrier in preterm infants [33]. Conversely, some studies have reported the association between an increased risk of NEC (Bell’s stage ≥ I) and antenatal glucocorticoid exposure [23]. Additionally, another trial revealed an increased NEC (Bell’s stage ≥ I) incidence with antenatal betamethasone administered every 12 h [34]. Another study found that exposure to antenatal corticosteroids was linked to a two-fold increase in the risk of NEC (stage unknown) [35].

#### 4.2.3. Strategies for Risk Mitigation and Exploration of Safer Alternatives

The use of corticosteroids for premature infants offers advantages but is accompanied by the risk of gastrointestinal issues. Although early (<8 days after birth) dexamethasone administration reduces the outcomes of death and BPD, the associated risk of gastrointestinal issues warrants caution. In contrast, early low-dose (1 mg/kg/day) hydrocortisone seems to be beneficial for survival without BPD, with fewer gastrointestinal complications [36]. However, concurrent NSAID treatment should be avoided with early hydrocortisone administration to minimize the risk of gastrointestinal perforation.

### 4.3. H2 Blockers

H2 blockers can be used to successfully manage gastric bleeding in infants [37], particularly VLBW infants treated with indomethacin or steroids, which are associated with an increased risk of gastrointestinal perforation [38]. Administering H2 blockers during such interventions could introduce an additional NEC risk [39], thereby compounding the existing risks associated with indomethacin and steroid use (Table 2).

#### 4.3.1. Mechanisms Underlying H2 Blockers and NEC Development

The alteration of gastric acidity, which is an important non-immune defense against infections, is crucial [40]. H2 blockers increase gastric pH, thereby hampering the elimination of ingested pathogens and potentially increasing susceptibility to gastrointestinal infections, particularly those caused by Gram-negative bacteria [41]. Furthermore, the use of H2 blockers may disrupt the delicate balance of microbial communities in the gut of preterm infants. Bacterial colonization and translocation are key components in NEC development. Moreover, H2 blockers directly impact intestinal motility and contractility [42], potentially aggravating the pathogenesis of NEC.

#### 4.3.2. Clinical Insights Regarding H2 Blockers and NEC

A case–control study by Guillet et al. highlighted a significant association between the use of H2 blockers and NEC (Bell’s stage ≥ II) for VLBW infants [39]. Similarly, a multicenter study by Terrin et al. revealed a 6.6-fold higher risk of NEC (Bell’s stage > II) for VLBW infants treated with ranitidine compared to that for controls [43]. A systematic review and meta-analysis by Santos et al. underscored the substantial links between H2 blockers and NEC (Bell’s stage ≥ II) [44]. However, a retrospective study by Singh et al. found no significant difference in the NEC (all Bell’s stages and Bell’s stage ≥ II) incidence of VLBW infants exposed to H2 blockers and that of those not exposed to H2 blockers [45]. Another retrospective study by Santana et al. found no significant association between ranitidine use and severe NEC (Bell’s stage ≥ II); however, ranitidine was associated with an increased risk of infection [46].

#### 4.3.3. Strategies for Risk Mitigation and Exploration of Safer Alternatives

H2 blockers are effective for preventing and treating NSAID-induced gastrointestinal damage [47]. A randomized, clinical trial demonstrated the efficacy of H2 blockers for reducing gastric bleeding or perforation caused by dexamethasone therapy [48]. The duration of NSAID-induced or steroid-induced gastrointestinal damage and the necessity for prolonged use of H2 blockers remain uncertain. Guillet et al. reported that NEC was observed an average of 19 days after treatment [39]. However, the high frequency of prophylactic therapy comprising H2 blockers (up to 72%) has raised concerns. Precise use of H2 blockers for preterm infants is essential to the effective reduction in the incidence of NEC.

### 4.4. Doxapram

Doxapram is a respiratory stimulant used to treat persistent apnea of prematurity, which poses a risk of insufficient brain development [49]. It stimulates both peripheral and central chemoreceptors, thereby enhancing ventilation and oxygenation [50]. Because the use of noninvasive respiratory support as an alternative to mechanical ventilation for VLBW infants is currently preferred, and because the importance of pharmaceutical therapy for apnea has increased, the role of doxapram has become significant, especially when methylxanthines (caffeine and aminophylline) are ineffective [51]. Nevertheless, the potential relationship of doxapram with NEC underscores the need for caution (Table 2).

#### 4.4.1. Mechanisms Underlying Doxapram and NEC Development

Although not fully elucidated, the following reasons for doxapram-induced gastrointestinal disturbances have been suggested: gastric perforation can be caused by the effect of doxapram on gastric acid hypersecretion [52,53]; doxapram suppresses intestinal smooth muscle contraction and high doses might disrupt intestinal transit, thereby potentially fostering NEC development [54]; and doxapram can alter the renal blood flow and systemic circulation, thereby influencing the intestinal blood flow and increasing the risk of NEC [55].

#### 4.4.2. Clinical Insights Regarding Doxapram and NEC

In Japan, because of the serious side-effects of doxapram, including NEC (Bell’s stage II) and gastric perforation [52,53], conventional dosing was contraindicated for neonates in 1995. The positive correlation between serum doxapram concentrations and adverse effects has prompted the exploration of lower doses [56]. Reduced-dose doxapram has demonstrated efficacy with fewer adverse effects [57]. Consequently, in 2015, doxapram was approved in Japan for the treatment of apnea of prematurity that is unresponsive to methylxanthines. However, cases of NEC (Bell’s stage II) related to low-dose doxapram use have been reported in Japan [58].

#### 4.4.3. Strategies for Risk Mitigation

If apnea persists despite methylxanthine administration, then doxapram should be considered before resorting to endotracheal intubation and mechanical ventilation. Internationally, the dosage of doxapram ranges from 0.2 to 5.0 mg/kg/h [59,60], with documented dose–response relationships [61]. Adverse effects are observed more commonly with higher doses (1.0–2.5 mg/kg/h) [59]. In Japan, to minimize adverse effects, doxapram is administered with caffeine or aminophylline using an initial intravenous infusion of 1.5 mg/kg over the course of 1 h, followed by a continuous intravenous infusion of 0.2 mg/kg/h. However, when that dose is ineffective, dose escalation to 0.4 mg/kg/h is considered.

### 4.5. Glycerin Enemas

Glycerin enemas offer a means of expediting meconium evacuation, thereby potentially leading to a faster transition to enteral feeding and improved clinical outcomes for premature infants. Shim et al. reported earlier full enteral feeding for infants who received routine glycerin enemas, particularly those with extremely low birth weight [62]. However, concerns regarding the association between glycerin enema use and the risk of NEC necessitate careful consideration of this approach (Table 2).

#### 4.5.1. Mechanisms Underlying Glycerin Enema Use and NEC Development

Glycerin enema use may affect the gut microbiota, which is crucial to intestinal health and immune regulation. Upsetting the microbial balance directly or through procedure-related stress may contribute to NEC development. Mechanical stimulation during enema administration may alter gut motility and blood flow, thereby potentially compromising the integrity of the mucosal barrier and increasing the susceptibility to inflammation and NEC.

#### 4.5.2. Clinical Insights Regarding Glycerin Enema Use and NEC

A systematic review and meta-analysis by Livingston et al. suggested a possible association between glycerin enema use and NEC (all Bell’s stages and Bell’s stage ≥II) development in premature infants [63]. Although enema use was correlated with an earlier initiation of stool passage and meconium evacuation, an increased NEC risk was observed. Conversely, a Cochrane review showed that prophylactic glycerin laxatives did not affect the incidence of NEC (any stage) [64]. A national survey by Gross et al. found no difference in the incidence of NEC (stage unknown) among glycerin enema users and non-users [65]. A meta-analysis by Burchard et al. also suggested that glycerin enemas have no definitive effects on NEC (stage unknown); however, they were associated with earlier meconium evacuation [66].

#### 4.5.3. Strategies for Risk Mitigation and Exploration of Safer Alternatives

The use of glycerin enemas to expedite meconium evacuation and enteral feeding has contradictory effects on the risk of NEC. Therefore, the evidence quality may be low. Whether the enema-associated NEC risk stems from its composition remains uncertain. A meta-analysis encompassing different enemas did not find an increased risk of NEC [67]. However, one study reported NEC rates of 23% for saline enema recipients and 3% for controls [68]. Recent studies have not reported severe NEC cases involving infants treated with normal saline enemas during the initial postnatal period [69,70].

### 4.6. Antibiotics

Antibiotics are frequently prescribed to prevent or treat infections in premature infants. Additionally, antibiotics are often administered empirically to preterm infants to address the critical nature of early-onset sepsis. Consequently, more than 75% of VLBW infants are exposed to empirical antibiotics [71]. Although antibiotics have a vital role in the management of bacterial sepsis, their inappropriate or excessive use may inadvertently increase the risk of serious outcomes, including NEC (Table 2).

#### 4.6.1. Mechanisms Underlying Antibiotics and NEC Development

Antibiotics decrease bloodstream infections by delaying colonization and reducing bacterial loads in the intestinal mucosa and epithelial border [72]. They also influence the commensal intestinal microbiota, which is pivotal for intestinal health, immune regulation, and pathogen resistance. Early empirical antibiotic exposure is associated with enhanced mucosal integrity and a reduced inflammatory response, thereby suggesting its potential to protect the intestines of preterm infants through immune modulation associated with early microbiota colonization [73] and the delayed colonization of pathogenic bacteria [74]. However, disrupting this delicate microbial balance can lead to an increase in pathogenic bacteria and a decrease in beneficial bacteria, thereby fostering an environment conducive to NEC development. The antibiotic duration also affects neonatal intestinal colonization, with prolonged therapy potentially upsetting the microbiota balance toward pathogens, thereby increasing the risk of NEC [75]. Moreover, antibiotics may alter the integrity of the intestinal mucosal barrier, possibly affecting the gut protection mechanisms [29].

#### 4.6.2. Clinical Insights Regarding Antibiotics and NEC

During a prospective multicenter cohort study by Dierikx et al., the group without early empirical antibiotic exposure had a higher adjusted risk of NEC (Bell’s stage ≥II) development [76]. However, prolonged early empirical antibiotic exposure may offset this benefit by disrupting microbial colonization [77]. Although empirical therapy is typically ceased upon the receipt of negative blood culture results after 48–72 h, the undertreatment of clinical sepsis often results in prolonged courses [78]. Zhu et al. linked prolonged antibiotic durations to the NEC (Bell’s stage ≥ II) risk for VLBW infants, thereby implying that the duration of the initial empirical therapy might be a risk factor for NEC [79]. Similarly, many studies have reported an increased risk of NEC with prolonged (≥5 days) use of the initial antibiotics [80,81,82,83]. Conversely, Greenberg et al. found no association between prolonged antibiotic use and NEC (Bell’s stage ≥ II) or death [84]. The NEOMUNE study, which involved 2831 VLBW infants, showed no significant difference in the NEC (Bell’s stage ≥ II) incidence of the short (≤72 h) antibiotic treatment group and that of the prolonged (>72 h) antibiotic treatment group [74]. Nevertheless, that study reported a lower incidence of NEC (3.9%) among infants after early antibiotic exposure than among non-exposed infants (9%).

#### 4.6.3. Strategies for Risk Mitigation

Although early empirical antibiotics reduce the risk of NEC, their prolonged use may increase the risk of NEC in VLBW infants. If laboratory results and clinical trends do not indicate bacterial infections, then the discontinuation of antibiotic therapy after 72 h could be considered. Moreover, the use of antibiotics for premature infants without ongoing infection leads to potential short-term and long-term consequences for the development of the microbial structure and immune system [83].

## 5. Clinical Dilemma Involving Other Treatments and the Risk of NEC

### 5.1. Blood Transfusions

Blood transfusions are integral to managing anemia and improving the oxygen-carrying capacity of premature infants. More than half of VLBW infants receive one or more transfusions during hospitalization [85]. Although these treatments are lifesaving, they have been linked to an increased risk of NEC (Table 2). Transfusion-associated NEC (TANEC) is associated with the occurrence of NEC (Bell’s stage ≥ II or III) within 48 h after red blood cell (RBC) transfusion [86]. According to a review report by Khashu et al. [87], several studies have suggested that TANEC occurred later than NEC unrelated to transfusion in infants born at earlier gestational ages; TANEC occurred 3–5 weeks after birth, whereas NEC unrelated to transfusions occurred 1–3 weeks after birth. Elabiad et al. reported that VLBW infants with NEC (Bell’s stage ≥ II) who received RBC transfusions had a lower postmenstrual age at the time of NEC than VLBW infants who did not [88].

#### 5.1.1. Mechanisms Underlying Blood Transfusions and NEC

Several mechanisms may contribute to TANEC development, including a compromised gut blood flow resulting from severe anemia, the exposure to immune triggers in transfused blood, and ischemia–reperfusion injury [89,90]. Observational studies have linked severe anemia (hematocrit level ≤ 25% or ≤8 g/dL) to an increased risk of TANEC for preterm infants [91,92]. Animal models have suggested that endogenous vasoactive mediators and inflammation have roles in TANEC (because of the upregulation of TLR4 and activation of pro-inflammatory macrophages) [93].

#### 5.1.2. Clinical Evidence of Blood Transfusions and NEC

TANEC has been estimated to account for 20–35% of NEC cases [94]. A meta-analysis associated RBC transfusions with a doubled risk of NEC (stage unknown) [95]. Conversely, some studies have shown no association between RBC transfusions and NEC (Bell’s stage ≥ II) [91,96]; furthermore, some studies have indicated that RBC transfusions have protective effects [97,98]. Feeding practices during transfusions may cause complications; however, the effects on NEC remain uncertain [99,100].

#### 5.1.3. Strategies for Risk Mitigation

The optimal hemoglobin thresholds, safe RBC product characteristics, and feeding protocols during transfusions remain unresolved. However, advanced monitoring methods, such as near-infrared spectroscopy, are promising [101]. Standardized feeding protocols may reduce the risk of NEC, but the implications of feeding during RBC transfusions remain unknown.

### 5.2. Probiotics

Probiotics, which are live microorganisms recognized by the World Health Organization as having health benefits when present in adequate amounts [102], have been studied to determine their potential to reduce the risk of NEC in premature infants [103]. However, a fundamental dilemma has arisen because the most vulnerable neonates who could benefit from probiotics are also prone to sepsis caused by the same microorganisms.

#### 5.2.1. Mechanisms Underlying the Actions of Probiotics and NEC Prevention

Probiotics potentially safeguard against NEC by reinforcing the intestinal barrier against bacteria and toxins, shaping the host response to microbial products, enhancing the mucosal response to immunoglobulin A, producing bactericidal substances, and outcompeting potential pathogens [104,105].

#### 5.2.2. Clinical Insights Regarding Probiotics and NEC Prevention

A systematic review of 30 randomized controlled trials and 14 observational studies suggested that probiotics can prevent severe NEC (Bell’s stage ≥ II) in VLBW infants [106]. Notably, *Lactobacillus rhamnosus* GG and *Bifidobacterium lactis* alone significantly reduced the incidence of severe NEC [106]. Both the mother’s own milk and donor human milk are known to decrease the incidence of NEC [6]. Sato et al. showed that a diet exclusively comprising human milk and daily probiotic supplementation was associated with a decreased incidence of NEC (Bell’s stage ≥ I) in VLBW infants [107]. Moreover, Sharpe et al. reported that the incidence of NEC (Bell’s stage ≥ II) in very preterm infants was lower for those whose diet comprised probiotics and pasteurized donor human milk; however, this incidence was not statistically significant [108]. However, some studies have not consistently shown significant protection against NEC with probiotics [109,110]. In a preterm pig model, Cilieborg et al. observed an increased NEC incidence as well as an increased NEC severity with probiotics, possibly because of the increased proinflammatory cytokine expression [111].

#### 5.2.3. Probiotics and Adverse Effects

Although the administration of live microorganisms to immature infants raises concerns about probiotic-associated sepsis and bacteremia, molecular techniques have identified cases of sepsis related to *Saccharomyces cerevisiae*, *Bifidobacterium* species, and *Lactobacillus* species in term and preterm infants [112,113,114,115]. The risk of sepsis does not appear to be related to the dose of probiotics or their duration of use [114]. Successful adhesion and colonization of the intestinal mucosa are vital to the actions of probiotics; however, impaired gut integrity can lead to translocation and bacteremia [116]. Gut ischemia can worsen this risk for preterm infants. As reported by Chiang et al., biofilm formation on central venous catheters can contribute to persistent probiotic-associated bacteremia [113].

#### 5.2.4. Balancing the Risks and Benefits

Considering the low risk of sepsis associated with probiotics, the appropriate use of probiotics should not be discouraged. However, there is no consensus regarding the selection, dosage, or treatment duration of probiotics. The American Academy of Pediatrics cautions against the routine administration of probiotics to preterm infants, especially those with extremely low birth weight, because of the lack of approval from the Food and Drug Administration [117]. The American Academy of Pediatrics recommends obtaining informed parental consent after discussing the risks and benefits of administering probiotics.

## 6. Conclusions

This review underscores the delicate balance between enhanced outcomes of VLBW infants and the associated risk of NEC. Physicians must handle the dilemma of weighing the potential benefits of treatment for VLBW infants against the increased risk of NEC. The existing evidence regarding the treatments for and the NEC risk of VLBW infants is diverse, and variations in study designs and patient cohorts potentially influence the outcomes. Moreover, confounding factors such as underlying medical conditions and concurrent treatments can further complicate the NEC risk assessment. Modifying treatment plans because of NEC concerns during the management of VLBW infants is challenging. Because of the contradictory evidence regarding the treatments for and the NEC risk of this population, physicians should adopt an individualized approach for each case. Factors such as gestational age, clinical condition, and NEC risk indicators should guide treatment decisions. Careful vigilance for early signs of NEC during treatment is crucial, as are proactive preventive measures to mitigate the NEC risk. When interpreting NEC studies and their results, it is important to consider their diagnostic methods and staging, especially those of older reports. However, their exploration during a narrative study can be challenging. Future research is imperative to unravel the intricate interactions between the treatments for and the NEC risk of VLBW infants.

## Figures and Tables

**Table 1:** Factors associated with the risk of necrotizing enterocolitis.

Main Risk Factors
Prematurity
Low birth weight
Formula feeding
Intestinal dysbiosis
Maternal Factors
Increased body mass index
Intrahepatic cholestasis
Smoking
Cocaine use
Chorioamnionitis
Placenta abruption
Preeclampsia
Antenatal antibiotic use
Prolonged rupture of membranes
Cesarean delivery
Fetal factors
Genetic predisposition
Intrauterine growth restriction
Non-reassuring fetal state
Lack of antenatal steroids
Neonatal factors
Hypoxia
Congenital heart disease
Gastrointestinal anomaly
Patent ductus arteriosus
Anemia
Polycythemia
Treatment administered to very low-birth-weight infants
Medications
Indomethacin
Corticosteroids
Histamine-2 receptor blockers
Doxapram
Glycerin enema
Antibiotics
Blood transfusions including exchange transfusion
Umbilical catheterization

**Table 2:** Clinical benefits and risks of treatments for very low-birth-weight infants.

Treatment	Indication	Clinical Benefits	Potential Risks of NEC
Indomethacin	To prevent or treat symptomatic PDA	Closes the PDA	Harmful effects on blood flow to the intestines and reduces intestinal perfusion
Corticosteroids	To treat respiratory distress in BPD	Improves lung function and reduces BPD severity	Harmful effects on intestinal immune defense, motility, circulation, and barrier function
Histamine-2 receptor blockers	Gastric bleeding (e.g., bloody nasogastric tube aspirates)	Protects the delicate gastrointestinal mucosa by decreasing gastric acid secretion	Harmful effects on gastrointestinal tract host defense caused by increased gastric pH
Doxapram	Persistent apnea unresponsive to methylxanthines	Stimulates chemoreceptors, enhances ventilation and oxygenation	Gastrointestinal disturbance caused by gastric acid hypersecretion, intestinal smooth muscle contraction, and intestinal blood flow change
Glycerin enema	To promote meconium evacuation and accelerate stool passage	Reduces the risk of meconium-related complications and facilitates bowel movements	Damages bowel epithelial cells, influences the composition of the gut microbiota, and changes gut motility and intestinal blood flow
Antibiotics	To prevent and treat bacterial infections	Controls bacterial infections	Overuse or misuse can lead to drug resistance and disrupt the gut microbiome
Blood transfusion	Anemia or other blood-related conditions	Corrects anemia and improves the oxygen-carrying capacity	Changes gut perfusion and the immune response
Probiotics	To improve the gut microbiome	Reduces the risk of NEC by promoting healthy gut flora and enhancing the gut barrier function	Cause infections, particularly in critically ill or immunocompromised infants

NEC, necrotizing enterocolitis; PDA, patent ductus arteriosus; BPD, bronchopulmonary dysplasia.

## Data Availability

Not applicable.
